# Active-State Model of a Dopamine D_2_ Receptor - Gα_i_ Complex Stabilized by Aripiprazole-Type Partial Agonists

**DOI:** 10.1371/journal.pone.0100069

**Published:** 2014-06-16

**Authors:** Ralf C. Kling, Nuska Tschammer, Harald Lanig, Timothy Clark, Peter Gmeiner

**Affiliations:** 1 Department of Chemistry and Pharmacy, Emil Fischer Center, Friedrich Alexander University, Erlangen, Germany; 2 Department of Chemistry and Pharmacy, Computer Chemistry Center, Friedrich Alexander University, Erlangen, Germany; 3 Central Institute for Scientific Computing, Friedrich Alexander University, Erlangen, Germany; 4 Centre for Molecular Design, University of Portsmouth, King Henry Building, Portsmouth, United Kingdom; Medical School of Hannover, Germany

## Abstract

Partial agonists exhibit a submaximal capacity to enhance the coupling of one receptor to an intracellular binding partner. Although a multitude of studies have reported different ligand-specific conformations for a given receptor, little is known about the mechanism by which different receptor conformations are connected to the capacity to activate the coupling to G-proteins. We have now performed molecular-dynamics simulations employing our recently described active-state homology model of the dopamine D_2_ receptor-Gα_i_ protein-complex coupled to the partial agonists aripiprazole and FAUC350, in order to understand the structural determinants of partial agonism better. We have compared our findings with our model of the D2R-Gα_i_-complex in the presence of the full agonist dopamine. The two partial agonists are capable of inducing different conformations of important structural motifs, including the extracellular loop regions, the binding pocket and, in particular, intracellular G-protein-binding domains. As G-protein-coupling to certain intracellular epitopes of the receptor is considered the key step of allosterically triggered nucleotide-exchange, it is tempting to assume that impaired coupling between the receptor and the G-protein caused by distinct ligand-specific conformations is a major determinant of partial agonist efficacy.

## Introduction

G protein-coupled receptors (GPCRs) constitute an important class of membrane-bound glycoproteins that participate in the regulation of various physiological processes including heartbeat, breathing and our senses of vision, smell and taste [1]. By transmitting extracellular stimuli to the inside of the cell, GPCRs serve as linker molecules that connect ligand binding to the coupling of intracellular binding partners including G proteins or β-arrestins [2]. In its canonical signaling pathway, the activated, neurotransmitter/hormone-occupied receptor couples to G proteins, thereby inducing conformational changes that give rise to nucleotide-exchange and culminate in various functional responses [3]–[6]. The efficacy of a given ligand refers to its capacity to enhance the coupling of one receptor to a particular intracellular effector protein, thereby inducing quantifiable cellular responses [7], [8]. Depending on the extent of their functional response, ligands can be classified as neutral antagonists or inverse agonists, neither of which stimulates receptor activation, full agonists, which cause a cellular response that strongly resembles that of the endogenous ligand, and partial agonists, which exhibit submaximal effects even at saturating concentrations.

Partial agonism at dopamine D_2_ receptors (D2R) has been suggested to exert beneficial effects on schizophrenia, a chronic mental illness characterized by hypo- and hyperfunctions in monoamine neurotransmitter systems, including the mesolimbic and mesocortical dopaminergic pathways [9]. The dopamine receptor partial agonists aripiprazole and the drug candidate cariprazine represent promising options for the treatment of schizophrenia [10]–[12] because of their stabilizing effect on monoamine pathways, especially the dopaminergic pathways, and their atypical antipsychotic effect.

Understanding the molecular basis of partial agonism requires detailed insight into the impact of ligands with different efficacies on the conformation of a given receptor or receptor-effector complex. Indeed, earlier studies that explored the origin of partial agonism revealed a ligand-dependent modulation of micro-switches important for receptor activation [13], [14] and specific ligand-specific conformations within receptor epitopes that include the orthosteric binding pocket, the extracellular loop (EL) region and areas of receptor-G protein coupling [14]–[20]. However, a structural study based on an active-state receptor coupled to an intracellular binding partner able to link ligand-specific receptor conformations to the capacity to activate a given effector has not yet been reported.

Significant progress in the field of GPCR-crystallography led the way to the elucidation of the crystal structure of the ternary β_2_ adrenergic receptor G_s_ protein complex [21], which provides an unprecedented framework for the investigation of ligand-induced receptor conformations and of receptor G protein interactions. Using this experimental structure as a template, we were able to generate active-state homology models of the dopamine D_2_ receptor (D2R) in complex with Gα_i_ and the endogenous agonist dopamine. We used these models to explore the structural determinants of selective receptor-G protein coupling on the amino-acid level using microsecond molecular-dynamics (MD) simulations [22].

We now report long-term all-atom MD simulations of our D2R-Gα_i_ homology model designed to help understand the molecular basis of partial agonist activity better. The D2R-Gα_i_ model in a hydrated bilayer environment was coupled to the partial agonist aripiprazole and a structurally related 1,4-disubstituted aromatic piperazine (1,4-DAP) [23], [24], FAUC350, which has been shown to induce a strongly impaired modulation of cAMP accumulation while activating ERK1/2 phosphorylation with moderate efficacy [25]. In this work, we have investigated the impact of aripiprazole and FAUC350 on the conformation of the ternary D2R-Gα_i_-complex, focusing on the shape of the extracellular loop region, the binding pocket and receptor-G protein-binding epitopes and compared our findings with our homology model of the D2R-Gα_i_-complex in presence of the full agonist dopamine. As in our previous work [22], [26], [27], we have performed single long simulations, rather than multiple shorter ones. This strategy allows us to avoid missing conformational changes that occur with a characteristic induction period, which either may be inherent to the natural system or be caused by a parameterized kinetic or thermodynamic structural bias of the force field towards “native” (i.e. X-ray or homology) protein structures.

## Results/Discussion

### Molecular-dynamics Simulations of Ternary D2R-Gα_i_-complexes Coupled to Dopamine, Aripiprazole and FAUC350

MD simulations with the ternary dopamine D2R-Gα_i_ complex revealed a high degree of conformational flexibility for the χ1-angle of His393^6.55^ (atoms: C-Cα-Cβ-Cγ) [22], a crucial residue for D2R activation [25], [28]. Despite this flexibility, a dihedral angle of around −60°, which was found almost continuously in the simulation denoted D2^Up^R (Table S1), turned out to be connected to favorable ligand-receptor, receptor-receptor and receptor-G protein-interactions (Figure S1). This simulation was used as a reference model with which to compare the simulations of D2R-Gα_i_ coupled to the partial agonists aripiprazole and FAUC350 (Tables S1 and S2). Starting from the existing membrane-inserted model, the dopamine ligand was removed and the partial agonists were docked. A more detailed description is given in the Materials and Methods section. The aripiprazole- and FAUC350-bound D2R-Gα_i_ complexes were subsequently submitted to energy minimization, equilibration and MD simulations runs of 800 ns and 500 ns, respectively. The G protein-moieties were more mobile than the receptor-units in both simulation systems (Figures S2 and S3). Nevertheless, the complexes showed no tendency for the G protein to dissociate from the receptor throughout the simulations (Figure S4).

### Aripiprazole and FAUC350 Open the Binding Pocket towards the Extracellular Surface

**Figure 1:**
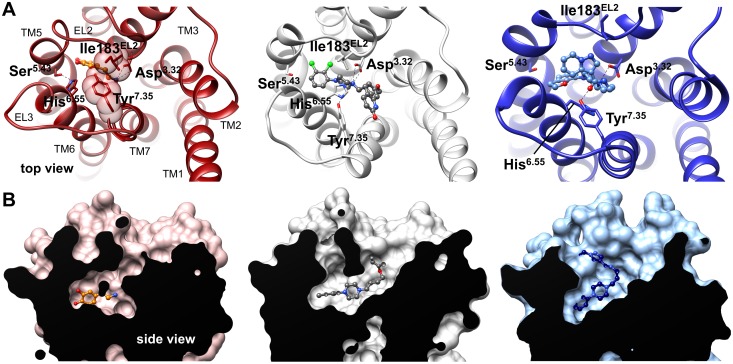
Ligand-specific conformations of the extracellular surface above the binding pocket. Representative snapshots of the dopamine- (left), the aripiprazole- (middle) and the FAUC350-complexes (right) are shown (average structures taken from 950–975 ns, 750–775 ns and 350–375 ns, respectively). (A) Extracellular view from the top into the binding pocket of the simulation systems. The ligands dopamine, aripiprazole and FAUC350 are highlighted in an orange, dark-grey and blue balls and sticks mode, respectively. The backbone of D2R is shown as ribbons, with important amino acids stabilizing the ligands indicated as sticks. (B) Side view into the binding pocket of the simulation systems, with a longitudinal section through the surface of D2R. Dopamine, aripiprazole and FAUC350 are represented as orange, dark-grey and blue balls and sticks, respectively. Compared to dopamine, both aripiprazole and FAUC350 open up the binding pocket towards the extracellular surface.

**Figure 2:**
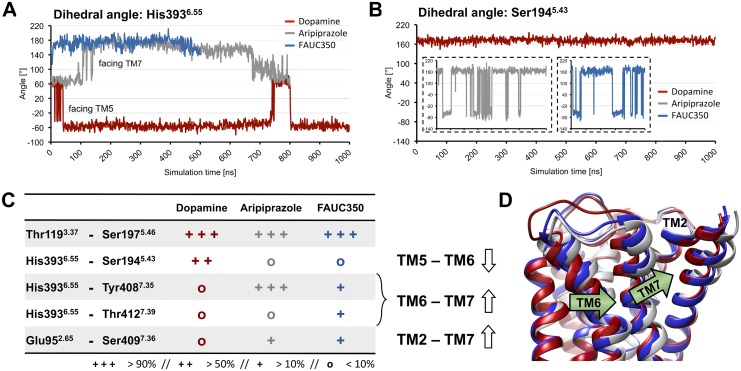
Investigation of dihedral angles and hydrogen-bond networks within the ligand binding pocket of the simulation systems. (A) Ligand-specific regulation of the dihedral angle of residue His393^6.55^ (atoms: C-Cα-Cβ-Cγ), depicted as red, grey and blue lines for the dopamine-, the aripiprazole- and the FAUC350-complexes, respectively. (B) The dihedral angle of residue Ser194^5.43^ (atoms: C-Cα-Cβ-Cγ) within the dopamine-simulation is shown as red lines. Unlike a constant value observed within the dopamine system, both aripiprazole (left insert) and FAUC350 (right insert) cause a greater flexibility of this dihedral angle. (C) Hydrogen-bond interactions between representative residues of helices TM2, TM3, TM5, TM6 and TM7. Aripiprazole (grey values) and FAUC350 (blue values) cause a ligand-specific modulation within interhelical networks in proximity to the binding pocket compared to dopamine (red values). (D) Representative snapshots of D2R within the dopamine-, the aripiprazole- and the FAUC350-complexes shown as red, grey and blue ribbons, respectively. The snapshots represent average structures taken from 950–975 ns (dopamine), 750–775 ns (aripiprazole) and 350–375 ns (FAUC350). The superposition of these structures visualizes ligand-specific changes within interhelical networks in proximity to the binding pocket. Helix movements are indicated with green arrows.

The models show that dopamine and the phenylpiperazine moieties of aripiprazole and FAUC350 occupy the same orthosteric binding pocket, thereby interacting with residues of transmembrane helices (TMs) 3, 5, 6, 7 and EL2. The linker moieties of the partial agonists show additional interactions with residues located at an extended binding pocket closer to the extracellular surface of the receptor (Figures S5 and S6). Depending on the ligand, we observed differently shaped structures above the binding pocket (Figure 1), which appeared to close around the full agonist dopamine so that EL2 and the extracellular tail of TM7 (measured as the distance between residues Ile183^EL2^ and Tyr^7.35^, Figure S7) are close to each other. In contrast, the partial agonists aripiprazole and, even more, FAUC350 opened up the binding pocket to the extracellular surface (Figure 1B). This opening is connected with the formation of a second binding pose for the partial agonist FAUC350 (Figures S2 and S5). Our observations are consistent with previous studies, which suggested ligand-specific conformations for EL2 and a regulatory function for this loop in receptor activation [29], [30]. The pronounced movement of the extracellular tail of TM7 towards EL2 within the dopamine-complex can be attributed to a ligand-induced hydrogen bond between Ser^5.43^ and His^6.55^, which shifts the side chain of His^6.55^ in the direction of TM5 and clears a space for the inward movement of Tyr^7.35^ (Figure 1). Unlike dopamine, aripiprazole and FAUC350 lead to a different dihedral angle (approximately 60° and, mainly, 180°) for His^6.55^ (Figures 1A and 2A), thereby increasing the distance between Tyr^7.35^ and EL2.

### Ligand-specific Interhelical Networks

Residue His^6.55^ was earlier found to play an important role in binding 1,4-DAPs, receptor activation and biased signaling [24], [25], [28]. The ligands dopamine, aripiprazole and FAUC350 can induce different conformations within the binding pocket of D2R by specifically adjusting the dihedral angle of His^6.55^ (Figure 2A). Thus, we attribute a key role to His^6.55^ in the ligand-dependent regulation of the binding pocket, which is consistent with experimental and computational reports. In addition to the ligand-specific conformational changes of His^6.55^, we captured differences in the interactions between the ligands and Ser^5.42^, Ser^5.43^ and Ser^5.46^ in TM5 (Figure S5). These residues have been shown to be crucial for the binding of different ligands, including catecholamines, and for an effective receptor-G protein-coupling [31], [32]. The full agonist dopamine formed stable hydrogen bonds to Ser^5.42^ and Ser^5.46^ and stabilized a conformation of Ser^5.43^, which facilitated hydrogen bonding to His^6.55^, while both aripiprazole and FAUC350 lacked hydrogen bonds to either serine residue and prevented the interaction between Ser^5.43^ and His^6.55^ (Figures 1 and S5). Moreover, the frequency with which the side chain of Ser^5.43^ pointed into the binding pocket was found to be reduced for aripiprazole and FAUC350 (Figure 2B). As receptor activation has been shown to be accompanied by such an inward movement of TM5-serines [33], [34], the hindered engagement of these amino acids observed for aripiprazole and FAUC350 is likely to result in reduced activation. In general, the ligands investigated exerted different effects on specific conformations of amino-acid networks within the orthosteric binding pocket of D2R. Whereas the full agonist dopamine stabilized interactions between TM5 and TM6 close to the binding pocket, aripiprazole and FAUC350 led to helical reorientations such that TM6 moved away from TM5 towards TM7, thereby strengthening interactions between TM6 and TM7 and between TM7 and TM2 (Figure 2C, D). In contrast, interactions between TM3 and TM5, measured as a hydrogen bond between Thr^3.37^ and Ser^5.46^, remained mostly unmodified (Figure 2C).

The predicted binding modes for the endogenous agonist dopamine and the two partial agonists aripiprazole and FAUC350 differ significantly from that of the antagonist eticlopride in the crystal structure of the closely related dopamine D_3_ receptor (D3R) [35] (Figure S8). The full agonist dopamine is located deeper within the binding pocket of D2R than eticlopride (Figure S8A). In this conformation, the ammonium nitrogen of dopamine is stabilized by an ionic interaction to Asp^3.32^ and a cation-π interaction to Phe^6.51^, thereby connecting TM3 and TM6 at residues that have been shown to be important for both ligand binding and receptor activation [24], [36]. This connection between two helices that are relevant for activation is prevented by eticlopride because its positively charged nitrogen atom is displaced by 3.4 Å compared its dopamine equivalent and its ethyl moiety helps shield the positive charge. Comparable but less pronounced displacements of the positively charged nitrogen atoms were observed in the partial agonist complexes (2.2 Å and 2.9 Å for aripiprazole and FAUC350, respectively) (Figure S8B and C). In these cases, it is still possible to connect helices TM3 and TM6 structurally, even though the conformation of Phe^6.51^ differs from that within the dopamine complex (and His^6.55^ helps stabilize the charged nitrogen atoms by cation-π interactions). These observations thus suggest a more general role for the positively charged nitrogen atom of (partial) agonists in stabilizing the active-state of D2R in contrast to its role in the binding mode of antagonists such as eticlopride. Further studies of N-substituted agonists are needed in order to explore the possible conformational influences of the structural connections around the protonated nitrogen on receptor activation.

### A Hydrophobic Network Connects the Ligand- and the G Protein-binding Pockets and is Regulated Differently by the Ligands

**Figure 3:**
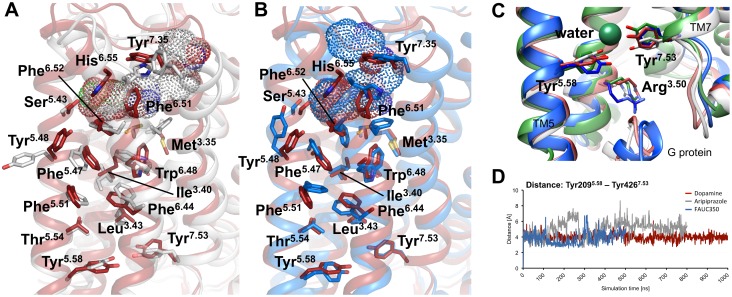
Hydrophobic network between the extracellular and the intracellular surface of D2R. The snapshots shown represent average structures taken from 950–975 ns (dopamine), 750–775 ns (aripiprazole) and 350–375 ns (FAUC350). (A, B) The backbone of D2R is shown as ribbons, important amino acids comprising the hydrophobic network are visualized as sticks. The ligands within their binding pockets are highlighted as dotted spheres. Superposition of representative snapshots taken from the dopamine- (red), the aripiprazole- (grey) and the FAUC350-simulations (blue) indicate ligand-specific conformations of residues within this hydrophobic network. (C) A water-mediated hydrogen bond between residues Tyr^5.58^ and Tyr^7.53^ (represented as sticks) of the crystal structure of β2AR bound to the ligand BI167107 and an intracellular nanobody (PDB-ID: 4LDE) is shown in green, with residue Arg^3.50^ forming the upper end of the G protein binding pocket. Additionally, representative snapshots of the dopamine- (red), the aripiprazole- (grey) and the FAUC350-simulations (blue) are superimposed with the crystal structure. (D) The distance between the hydroxyl groups of residues Tyr^5.58^ and Tyr^7.53^ within the dopamine-, the aripiprazole- and the FAUC350-complexes are shown as red, grey and blue lines, respectively.

Ligand-binding to the extracellular part of the receptor is connected to conformational changes on its intracellular side [37], which points to the existence of an allosteric communication path that transforms rather small changes within the orthosteric binding pocket into pronounced intracellular rearrangements [38]–[41]. Earlier studies have identified hydrophobic residues at the core of TM3, TM5, TM6 and TM7 to be involved in this signal propagation; key roles in receptor activation were attributed to the so called ‘transmission switch’, consisting of Ile^3.40^, Pro^5.50^ and Phe^6.44^ [39], and the ‘rotamer toggle switch’, centered around Trp^6.48^ [42], [43]. Consistent with these studies, our MD simulations depicted an allosteric communication network that links the ligand-binding pocket to the G protein-coupling domain (Figure 3A, B). Starting from distinct dihedral angles of His^6.55^ within the binding pocket, we observed ligand-specific conformational changes of individual residues of this network (Figure S9), including the aromatic amino acids Phe^6.44^, Trp^6.48^ and Phe^6.52^, which are known to be crucial for receptor activation [39], [40], [44], [45]. The lower end of this network is formed by the highly conserved residues Tyr^5.58^ and Tyr^7.53^ (Figure 3C), which were suggested to stabilize the active-state of the receptor via a water-mediated hydrogen-bond [46], [47]. In analogy, it was found that mutation of Tyr^5.58^ in rhodopsin is involved in allosteric coupling to EL2[48] and in a reduction in the capacity to activate transducing [49]. Whereas the hydrogen bond between Tyr^5.58^ and Tyr^7.53^ remained stable throughout the dopamine-simulation, aripiprazole and FAUC350 caused a larger fluctuation in the distance between these residues (Figure 3D).

### Full and Partial Agonists Influence the Conformation of G Protein-binding Epitopes of D2R Differently

**Figure 4:**
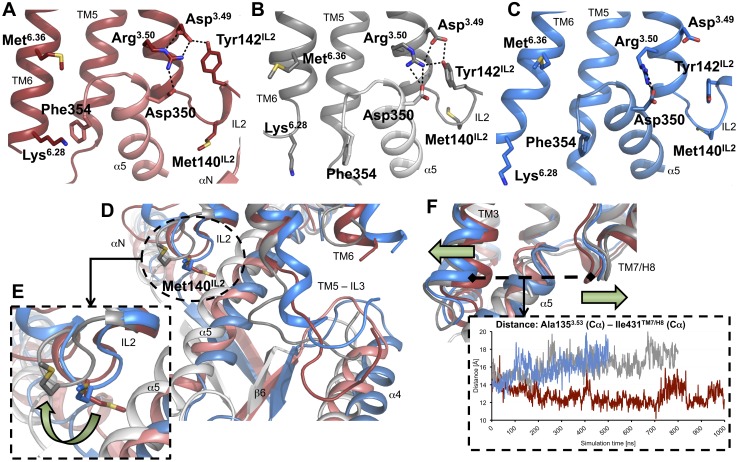
Crucial amino-acid interactions and conformational changes within G protein coupling domains. The backbone of D2R is shown as ribbons, whereas important amino acids are highlighted as sticks. The snapshots provided represent average structures taken from 950–975 ns (dopamine), 750–775 ns (aripiprazole) and 350–375 ns (FAUC350). (A–C) Crucial interactions between residues from the C-terminal part of Gα (Asp350, Phe354) and residues from the DRY-motif of TM3 (Asp^3.49^, Arg^3.50^), from IL2 (Met140, Tyr142) and from TM6 (Lys^6.28^, Met^6.36^) within representative snapshots of dopamine-, aripiprazole- and FAUC350-complexes are depicted in red, grey and blue, respectively. (D) A superposition of representative snapshots of dopamine-, aripiprazole- and FAUC350-complexes, represented in red, grey and blue, respectively, indicates conformational changes for residue Met140 of IL2 and for the N-terminal part of IL3. (E) Enlarged view of Figure 4d on the conformational changes of residue Met140 of IL2 within the simulation systems. A green arrow visualizes the movement of residue Met140. (F) A superposition of representative snapshots of dopamine-, aripiprazole- and FAUC350-complexes, represented in red, grey and blue, respectively, highlights the increasing distance of the intracellular part of TM3 and the junction of TM7 and H8, measured as the distance between the Cα-atoms of Ala135^3.53^ and Ile431 of TM7/H8 (dashed box).

**Figure 5:**
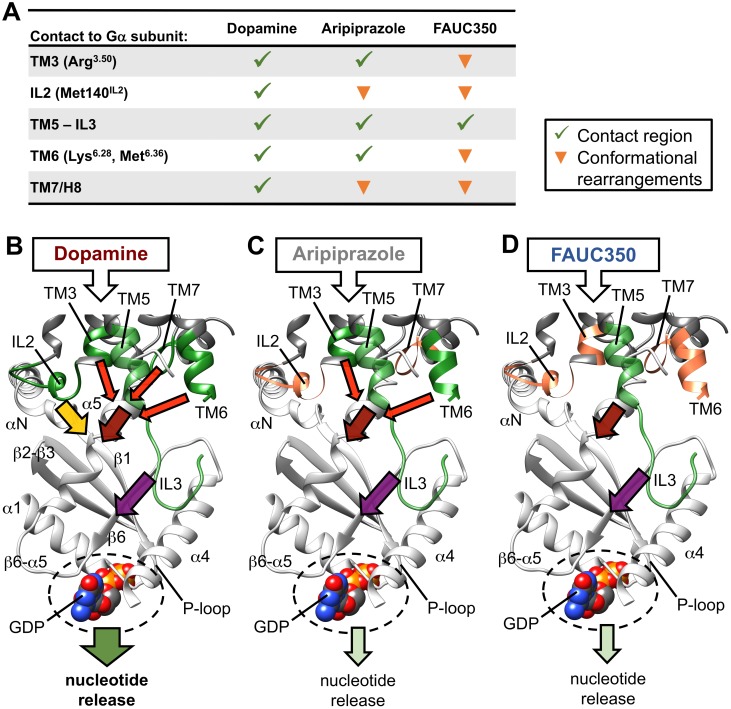
Conformational changes within G protein coupling domains and their supposed effect on nucleotide release. (A) A summary of certain conformations of G protein coupling epitopes of D2R observed within the simulation systems is shown. (B–D) One representative snapshot of the dopamine-complex, taken as an average structure from between 950–975ns, is used as a scaffold, in which to compare the effect of the ligands dopamine, aripiprazole and FAUC350 on the conformation of G protein coupling domains and thus on nucleotide release schematically. Areas of receptor-G protein coupling are shown as dark-grey and light-grey ribbons, respectively. The conformation of GDP has been taken from a superposition of the aforementioned snapshot with the crystal structure of ground-state Gα_i_ (PDB-ID: 1GP2). Stable contact regions of D2R are highlighted in green, conformational changes are indicated in orange. Colored arrows imply the supposed contribution of individual G protein coupling domains (contacts to α5: red, contacts to αN/β1: yellow, contacts to α4/β6: violet) on nucleotide release (green arrows).

Finally, ligand-specific conformational changes involve domains of receptor-G protein coupling. The crystal structure of a representative ternary signaling complex [21] provides us with a precise molecular understanding of the interactions between an activated receptor and its G protein. Numerous experimental studies indicated that some receptor-domains constitute critical determinants for the activation of G proteins, including intracellular loop 2 (IL2) [50], intracellular loop 3 (IL3, connected to the intracellular ends of TM5 and TM6) [51]–[54], the DRY-motif (located at the intracellular end of TM3) [55], [56] and the proximal part of helix 8 (H8) [57]. Consequently, we analyzed the impact of our ligands on the conformation of these domains and found that the partial agonists aripiprazole and FAUC350 induce similar receptor conformations. However, these structures differ significantly from the receptor-dopamine complex (Figures 4 and 5A). One of these differences refers to the conformation of Met140^IL2^, whose side chain formed contacts to the G protein in the dopamine simulation, but was directed away in the aripiprazole and FAUC350 complexes (Figures 4A–D and S10). Moreover, a computational alanine scanning analysis of this residue revealed an impaired stabilization of the receptor-G protein interface within the dopamine-complex, whereas the M140A mutation within the aripiprazole- and the FAUC350-complexes exhibited weaker effects on receptor-G protein coupling (Figure S10), indicating a less important role for Met140^IL2^ in stabilizing the receptor-G protein interface within the latter two simulation systems. These observations are consistent with experimental studies showing that mutation to alanine at the corresponding position of β2AR and the muscarinic receptors M1 and M3, resulting in a loss of interaction, was connected to a reduced capacity to active G proteins [50]. IL2 is coupled structurally to the highly conserved DRY-motif at the intracellular end of TM3 (Figure 4A–C), which is known to be involved in G protein coupling via Arg^3.50^ [55], [56]. In the case of the dopamine and aripiprazole complexes, although with a subtly rearranged architecture in the aripiprazole simulation due to a conformational change of IL2 around Met140^IL2^, Arg^3.50^ was stabilized by Asp^3.49^ and Tyr142^IL2^ and formed an ionic interaction to Asp350 of the C-terminal part of Gα_i_ (Figure 4A–C, Figure S11). This stabilizing triad was mainly broken in the FAUC350-simulation, resulting in a less stable salt bridge (Figure S11). In addition to the intracellular part of TM3, the C-terminus of Gα_i_ is surrounded by the intracellular ends of TM5 and TM6 (constituting the beginning and the end of IL3, respectively) and the junction of TM7 and H8. Contacts of the C-terminus of Gα_i_ to the intracellular domain of TM5 were maintained throughout all three simulations (but with conformational changes for the open end of the N-terminal part of IL3) (Figures 4D and S12). However, the junction of TM7 and H8 moved away from the C-terminus of Gα_i_ in the aripiprazole and the FAUC350 complexes (Figure 4F). Moreover, cation-π (Lys^6.28^/Phe354) and hydrophobic interactions (Met^6.36^) between residues of TM6 and the C-terminal part of Gα_i_ were reduced in the aripiprazole- and the FAUC350-binding models (Figure 4A–C and S13).

Taken together, the conformational changes described in the G protein-binding domains of D2R directly influenced the conformation of the interacting epitopes of Gα_i_, including the C-terminus, helices α4 and α5 and the loops αN/β1 and β2/β3 (Figure 4). As it was recently shown that receptor-catalyzed nucleotide exchange is transmitted via dynamic changes within the linker regions connecting the areas of receptor-G protein and nucleotide-G protein-coupling [3], [5], we hypothesize that a complete G protein activation requires specific intracellular receptor conformations, which can only be stabilized by a full agonist like dopamine (Figure 5A). Distinct partial agonist-induced differences in the way intracellular epitopes are shaped may lead to an impaired receptor-G protein coupling and thus modulate the extent of the functional response (Figure 5B). It is therefore tempting to assume that impaired receptor-G protein coupling due to distinct ligand-specific conformations is a major determinant of partial agonist efficacy.

## Conclusions

In summary, we have used representative homology models of ternary receptor-G protein-complexes as structural scaffolds to investigate the molecular basis of partial agonism. We were able to capture distinct ligand-specific conformations within a homology model of our recently described ternary D2R-Gα_i_-complex, which help explain the graded efficacy of 1,4-DAP partial agonists such as aripiprazole and FAUC350 in comparison to the full agonist dopamine.

However, ligand-induced structural changes may differ for other receptor-effector systems such as receptor-β-arrestin-complexes [58] or even complexes of receptors with other G protein subtypes. This consideration is also relevant for both aripiprazole and FAUC350, which have been shown to exhibit biased signaling properties at D2R with respect to the activation of G protein- or β-arrestin-pathways, to Gα_i_/Gα_o_-signaling, or the stimulation of ERK1/2 phosphorylation [25], [59], [60]. Therefore, future studies will be required, ideally based on atomistic templates, to sample the conformations of a certain receptor-effector-complex entirely in order to explore the molecular determinants of biased agonism.

One purpose of our work is to investigate the potential of long MD simulations for investigating complex biological processes, especially for GPCRs, for which experimental evidence is often sketchy. The simulations reported above are at the high end of what is possible today, appear inherently reasonable and offer rationalizations of experimental observations. We have concentrated on “hard” results (structures, persistent interactions) in the main text and have reported less well-founded data (e.g. MMPBSA results) in the Supporting Information in order to provide as reliable results as possible. However, the simulations are inherently stochastic (because of their starting velocities) and the force fields only moderately well tested for simulations of this length. In particular, our preferred strategy of using single long simulations is not without alternative.

Nevertheless, we believe that the simulations reported above are relevant for the real GPCR system and that they potentially provide new atomistic details that can now be tested experimentally.

## Materials and Methods

### Simulation Systems

The simulation systems contain our recently described active-state homology models of the dopamine D_2_ receptor (D2^Down^R and D2^Up^R, depending on the initial rotamer conformation of the side chain of residue His393^6.55^ in the D2R models) in complex with a nucleotide-free Gα_i1_-protein [22], which were based on the crystal structure of the β_2_-adrenergic receptor (β2AR) together with a heterotrimeric G_s_-protein [21]. In addition, the models are embedded in a hydrated membrane consisting of dioleoylphosphatidylcholine (DOPC) lipids and coupled to the ligands dopamine, aripiprazole or FAUC350 (Tables S1 and S2).

Simulation A and B (Table S1) refer to previously published simulations of 1 µs each [22]. For simulation A, we performed an additional 500 ns simulation run as described [22].

Simulation systems C and D (Table S1) were prepared as follows: The ligands aripiprazole and FAUC350 were geometry optimized by means of Gaussian 09 [61] at the HF/6–31(d,p) level (attributing a formal charge of +1). AutoDock Vina [62] was used to subsequently dock both ligands into a membrane-inserted conformation of simulation system B. The ligand dopamine was removed before the docking procedure. We applied a search space of 28×26×40 Å to ensure a complete coverage of the binding pocket. The ligands were subjected to the docking procedure using an exhaustiveness value of 32 and a randomly selected starting position. 20 conformations of each ligand were obtained and inspected manually. Based on the scoring function of AutoDock Vina and experimental data, we selected one final conformation for each ligand. Parameter topology and coordinate files for the docked complexes were build up using the tleap module of AMBER10 [63] and subsequently converted into GROMACS input files [64], [65]. We finally exchanged the coordinates of the ternary dopamine-D2^Up^R-Gα_i1_-complexes (system B) within the membrane-inserted simulation systems with those of the docked aripiprazole- and FAUC350-D2^Up^R-Gα_i1_-complexes. The final simulation systems contained 460 DOPC-lipids surrounding the proteins and 8 chlorine atoms for charge neutralization. In total, systems C and D consisted of 227,577 atoms (51,300 water molecules) and 227,571 atoms (51,298 water molecules), respectively.

The final simulation systems were submitted to energy minimization (2500 steps of steepest descent minimization), equilibration (10 ns) and production molecular-dynamics simulation runs (800 ns and 500 ns for system C and D, respectively) using the GROMACS simulation package [64] as described earlier [26]. For all simulations, the general AMBER force field (GAFF) [66] was used for the ligands and the DOPC molecules and the force field ff99SB [67] for the protein residues. The GAFF force field for the lipids has been validated extensively by the original authors [68] and in our earlier work [22], [26]. The SPC/E water model [69] was applied. Parameters for the ligands were assigned using antechamber [63] and charges were calculated using Gaussian 09[61] at the HF/6–31(d,p) level and the RESP procedure according to the literature [70]. A formal charge of +1 was defined for the ligands. Throughout the productive simulations, a force of 1.0 kcal mol^−1^ Å^−2^ was applied to the N-terminal part of the G-protein’s αN-helix as described previously [22].

### Data Analysis

We removed water and DOPC molecules for data analysis. The analysis of the trajectories was performed with the PTRAJ module of AMBER10 [63]. Calculation of the binding free energies was accomplished using MMPBSA. Py [71]. Figures were prepared using PyMOL [72] and Chimera [73].

## Supporting Information

Figure S1 **Analysis of the dopamine simulations A and B.** (A–B) Representative conformations of the binding pocket of D2R within the simulation systems A and B are shown in green and red, respectively. Residues stabilizing dopamine (shown as sticks) in its binding pocket are represented as sticks, the backbone of D2R is shown as ribbons. Whereas both hydroxyl groups of dopamine’s catechol moiety participate in stabilizing a hydrogen bond network comprised of residues Ser^5.42^, Ser^5.43^, Ser^5.46^ and His^6.55^ in simulation B, dopamine is forming only one stable hydrogen bond to residue Ser^5.42^ in simulation A. (C) A hydrogen-bond analysis between dopamine and residues occupying the binding pocket of D2R is provided. (D) The dihedral angle of residue His393^6.55^ (atoms: C-Cα-Cβ-Cγ) for simulation A and B is depicted as green and red lines, respectively. (E–F) Representative conformations of the intracellular part of D2R within the simulation systems A and B are shown in green and red, respectively. Important amino acids are visualized as sticks. A (water-mediated) hydrogen bond between residues Tyr^5.58^ and Tyr^7.53^ of D2R and a salt bridge between residue Arg^3.50^ of D2R and Asp350 of Gα was only observed within simulation B, but not within simulation A. (G) The distances between the hydroxyl groups of the tyrosines Tyr^5.58^ and Tyr^7.53^ of D2R are depicted as green and red lines, respectively. (H–I) Free energy of binding calculations have been performed for dopamine-D2R (H) and D2R-Gα_i_ (I) using the GBSA-Method. The values are shown as green and red lines for simulation A and B, respectively, and indicate, in both cases, more stable interactions within simulation B.
(TIFF)

Figure S2 **RMS-deviations of the simulation systems.** RMS-deviations for individual components of the simulation systems are shown. The ligands and the receptors are fitted on the Cα-atoms of the receptors, whereas the G proteins are fitted on the Cα-atoms of the G-proteins. (A) RMSD-values for the ligand dopamine, D2R and Gα_i_ are given in yellow, dark-red and salmon, respectively. (B) RMSD-values for the ligand aripiprazole, D2R and Gα_i_ are given in black, dark-grey and light-grey, respectively. (C) RMSD-values for the ligand FAUC350, D2R and Gα_i_ are given in turquoise, dark-blue and light-blue, respectively. The values for FAUC350 indicate the existence of two interconvertible ligand conformations.
(TIFF)

Figure S3 **Atomic fluctuations within the simulation systems.** Atomic fluctuations for the Cα-atoms of the dopamine- (A), the aripiprazole- (B) and the FAUC350-complex (C) are shown in red, grey and blue, respectively. The thick lines for receptors and G proteins refer to a fit on Cα-atoms of receptors and G proteins, respectively, whereas the thin lines represent the fluctuations of the G proteins fitted on the receptor moieties.
(TIFF)

Figure S4 **Distances between receptors and the C-termini of the G proteins.** Distances between the centers of mass of D2R and the C-terminus of Gα_i_ for the dopamine- (A), the aripiprazole- (B) and the FAUC350-complex (C) are shown in red, grey and blue, respectively.
(TIFF)

Figure S5 **The binding pocket of the simulation systems.** Side view into the binding pocket of the simulation systems. The backbone of D2R is shown as ribbons, important amino-acids stabilizing the conformation of the ligands are represented as sticks. (A) A representative snapshot of the conformation of dopamine (orange balls and sticks) within the binding pocket is shown. (B) A representative snapshot of the conformation of aripiprazole (dark-grey balls and sticks) within the binding pocket is visualized. (C, D) Representative snapshots of the conformation of FAUC350 within the binding pocket are highlighted, taken from within the last 20 ns of the simulation time (light-blue balls and sticks, C) and at 400 ns (blue balls and sticks, D).
(TIFF)

Figure S6 **Residues within the binding pocket of D2R interacting with the ligands.** A detailed contact analysis of residues within the binding pocket of the simulation systems interacting with the ligands is provided. An amino acid is considered as forming a contact to a ligand when at least one atom of the amino acid approaches at least one atom of the ligand closer than 3.5 Å. The contacts are investigated throughout the simulated time scales.
(TIFF)

Figure S7 **Distance between EL2 and the extracellular end of TM7.** The distances between the side chains of Ile183 of EL2 and Tyr408^7.35^ of TM7 for the dopamine-, the aripiprazole- and the FAUC350-complex are shown in red, grey and blue, respectively.
(TIFF)

Figure S8 **Comparison of the predicted binding modes of our agonists at D2R with the conformation of the antagonist eticlopride at D3R.** Side view into representative snapshots of the binding pockets of D2R and the crystal structure of D3R. The snapshots represent average structures taken from 950–975 ns (dopamine), 750–775 ns (aripiprazole) and 350–375 ns (FAUC350). The backbone of the receptors is shown as ribbons, the ligands and important amino acids (Asp^3.32^, Phe^6.51^ and His^6.55^) stabilizing their conformation are represented as sticks. The positively charged nitrogen atoms of the ligands are highlighted as blue balls, whereas the distances between these nitrogen atoms of the ligands are given in light pink. The figure shows an overlay of eticlopride (green) at D3R with dopamine (orange/red, A), aripiprazole (grey sticks, B) and FAUC350 (blue, C) at D2R.
(TIFF)

Figure S9 **Ligand-specific dihedral angles of representative residues comprising the hydrophobic network between the ligand and the G protein binding pockets.** (A–H) Dihedral angles (atoms: C-Cα-Cβ-Cγ) of important residues from the core of the hydrophobic network (Ile^3.40^, Tyr^5.48^, Phe^6.44^, Trp^6.48^, Phe^6.51^, Phe^6.52^, Tyr^7.35^, Tyr^7.53^), which connect the ligand and the G protein binding pockets of D2R, are shown as dark-red, dark-grey and dark-blue lines representing the dopamine-, the aripiprazole- and the FAUC350-complexes, respectively. In addition, the dihedral angle of residue Trp^6.48^ between atoms Cα-Cβ-Cγ-Cδ_2_ (D) is provided as light-red, light-grey and light-blue lines for the dopamine-, the aripiprazole- and the FAUC350-complexes, respectively.
(TIFF)

Figure S10 **Investigations on residue Met140 of IL2.** (A) A computational alanine scanning analysis for residue Met140 of IL2 is provided for the dopamine- (left), the aripiprazole- (middle) and the FAUC350-complexes (right). Whereas the M140A mutation within the dopamine-complex was connected to an impaired stabilization of the receptor-G protein interface, we observed weaker effects of this mutation within the aripiprazole- and the FAUC350-complexes indicating a less important role of Met140^IL2^ within the latter two simulation systems. (B) The distance between the side chain of Met140 of IL2 and the Cα-atom of Ile343 of α5 for the dopamine- (left), the aripiprazole- (middle) and the FAUC350-complexes (right) is shown. An increasing distance between these residues indicates a conformation of Met140 exhibiting reduced contacts towards the G protein.
(TIFF)

Figure S11 **Distances between individual residues of TM3, IL2 and the α5 helix of the G protein.** The distances between individual residues of TM3 (Asp^3.49^, Arg^3.50^), IL2 (Tyr142) and the α5 helix of the G protein (Asp350) are shown as red, grey and blue lines for the dopamine-, the aripiprazole- and the FAUC350-complexes, respectively. The distance between residues Arg^3.50^ and Asp350 of the G protein comprising a salt bridge (A), between residues Arg^3.50^ and Asp^3.49^ of TM3 (B), between residues Arg^3.50^ of TM3 and Tyr142 of IL2 (C) and between residues Asp^3.49^ of TM3 and Tyr142 of IL2 (D) is provided.
(TIFF)

Figure S12 **RMS-deviations and contact analysis of the TM5-IL3 region.** (A) RMS-deviations for TM5 of D2R within the simulation systems are shown as red, grey and blue lines for the dopamine-, the aripiprazole- and the FAUC350-complexes, respectively. The values attribute a low conformational flexibility to TM5. (B) RMS-deviations for the proximal part of IL3 of D2R within the simulation systems are shown as red, grey and blue lines for the dopamine-, the aripiprazole- and the FAUC350-complexes, respectively. The values indicate a high conformational flexibility for IL3. (C, D) A detailed contact analysis between residues of TM5 and IL3 of D2R interacting with residues of the G protein is provided. An amino acid is considered as forming a contact when at least one atom of one amino acid approaches at least one atom of a second amino acid closer than 3.5 Å. The contacts are investigated throughout the simulated time scales.
(TIFF)

Figure S13 **Investigation of TM6 residues Lys367^6.29^ and Met374^6.36^.** (A) The distances between TM6 residue Lys367^6.29^ of D2R and the C-terminal residue Phe354 of Gα are shown. (B) The distances between D2R residues Arg132^3.50^ and Met374^6.36^ are shown. Values are represented in red, grey and blue for the dopamine-, the aripiprazole- and the FAUC350-complex, respectively.
(TIFF)

Table S1 **Overview of the simulation systems and their simulated time scales.**
(DOCX)

Table S2 **Chemical structures of the ligands investigated.**
(DOCX)
