# Supplementary figures and images for: Active-State Model of a Dopamine D2 Receptor - Gαi Complex Stabilized by Aripiprazole-Type Partial Agonists
Source: PLoS One. 2014 Jun 16;9(6):e100069. doi: 10.1371/journal.pone.0100069 (PMC4059746; doi:10.1371/journal.pone.0100069)

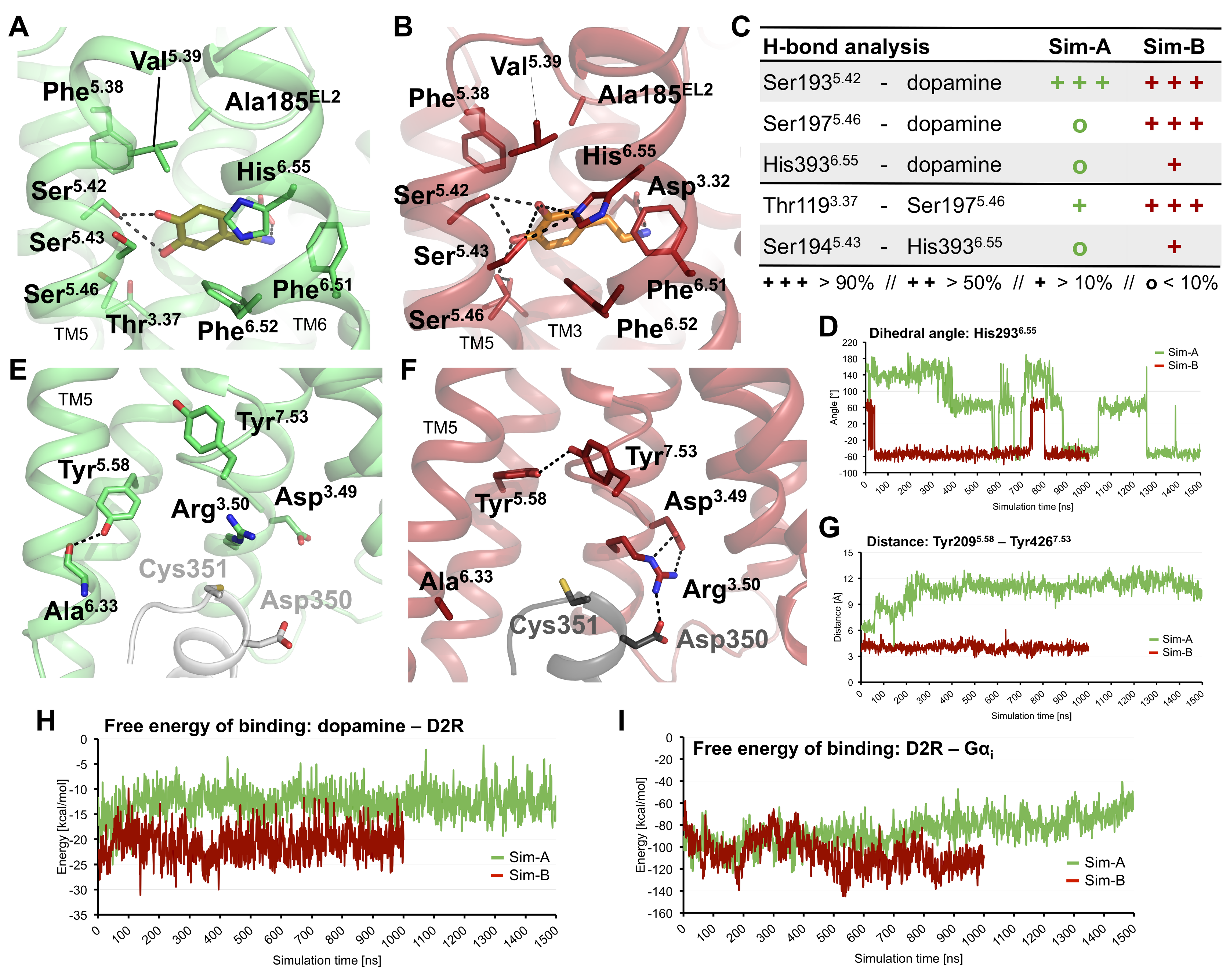

Supplement: Figure S1 — Analysis of the dopamine simulations A and B. (A–B) Representative conformations of the binding pocket of D2R within the simulation systems A and B are shown in green and red, respectively. Residues stabilizing dopamine (shown as sticks) in its binding pocket are represented as sticks, the backbone of D2R is shown as ribbons. Whereas both hydroxyl groups of dopamine’s catechol moiety participate in stabilizing a hydrogen bond network comprised of residues Ser5.42, Ser5.43, Ser5.46 and His6.55 in simulation B, dopamine is forming only one stable hydrogen bond to residue Ser5.42 in simulation A. (C) A hydrogen-bond analysis between dopamine and residues occupying the binding pocket of D2R is provided. (D) The dihedral angle of residue His3936.55 (atoms: C-Cα-Cβ-Cγ) for simulation A and B is depicted as green and red lines, respectively. (E–F) Representative conformations of the intracellular part of D2R within the simulation systems A and B are shown in green and red, respectively. Important amino acids are visualized as sticks. A (water-mediated) hydrogen bond between residues Tyr5.58 and Tyr7.53 of D2R and a salt bridge between residue Arg3.50 of D2R and Asp350 of Gα was only observed within simulation B, but not within simulation A. (G) The distances between the hydroxyl groups of the tyrosines Tyr5.58 and Tyr7.53 of D2R are depicted as green and red lines, respectively. (H–I) Free energy of binding calculations have been performed for dopamine-D2R (H) and D2R-Gαi (I) using the GBSA-Method. The values are shown as green and red lines for simulation A and B, respectively, and indicate, in both cases, more stable interactions within simulation B. (TIFF) [file pone.0100069.s001.tiff]

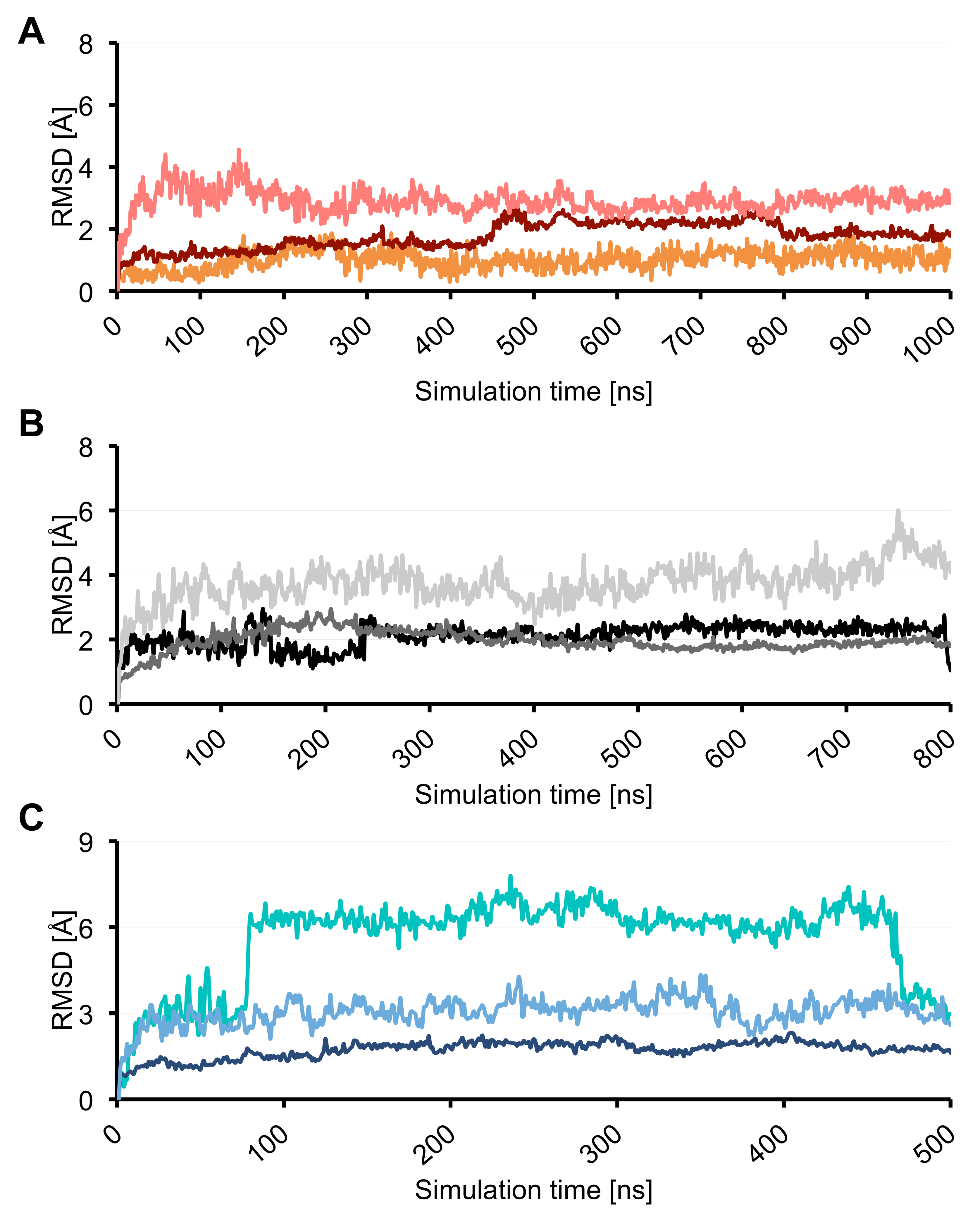

Supplement: Figure S2 — RMS-deviations of the simulation systems. RMS-deviations for individual components of the simulation systems are shown. The ligands and the receptors are fitted on the Cα-atoms of the receptors, whereas the G proteins are fitted on the Cα-atoms of the G-proteins. (A) RMSD-values for the ligand dopamine, D2R and Gαi are given in yellow, dark-red and salmon, respectively. (B) RMSD-values for the ligand aripiprazole, D2R and Gαi are given in black, dark-grey and light-grey, respectively. (C) RMSD-values for the ligand FAUC350, D2R and Gαi are given in turquoise, dark-blue and light-blue, respectively. The values for FAUC350 indicate the existence of two interconvertible ligand conformations. (TIFF) [file pone.0100069.s002.tiff]

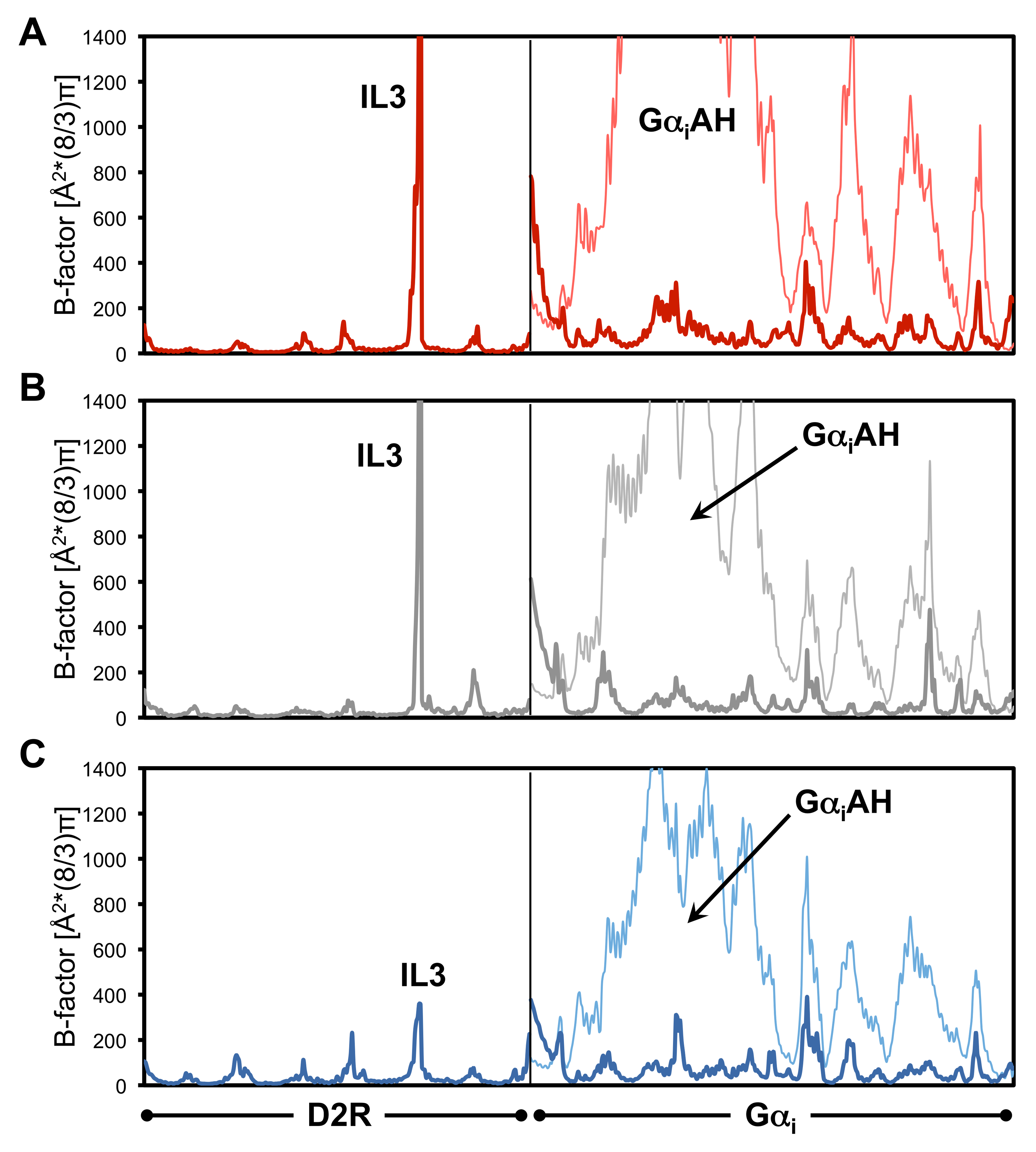

Supplement: Figure S3 — Atomic fluctuations within the simulation systems. Atomic fluctuations for the Cα-atoms of the dopamine- (A), the aripiprazole- (B) and the FAUC350-complex (C) are shown in red, grey and blue, respectively. The thick lines for receptors and G proteins refer to a fit on Cα-atoms of receptors and G proteins, respectively, whereas the thin lines represent the fluctuations of the G proteins fitted on the receptor moieties. (TIFF) [file pone.0100069.s003.tiff]

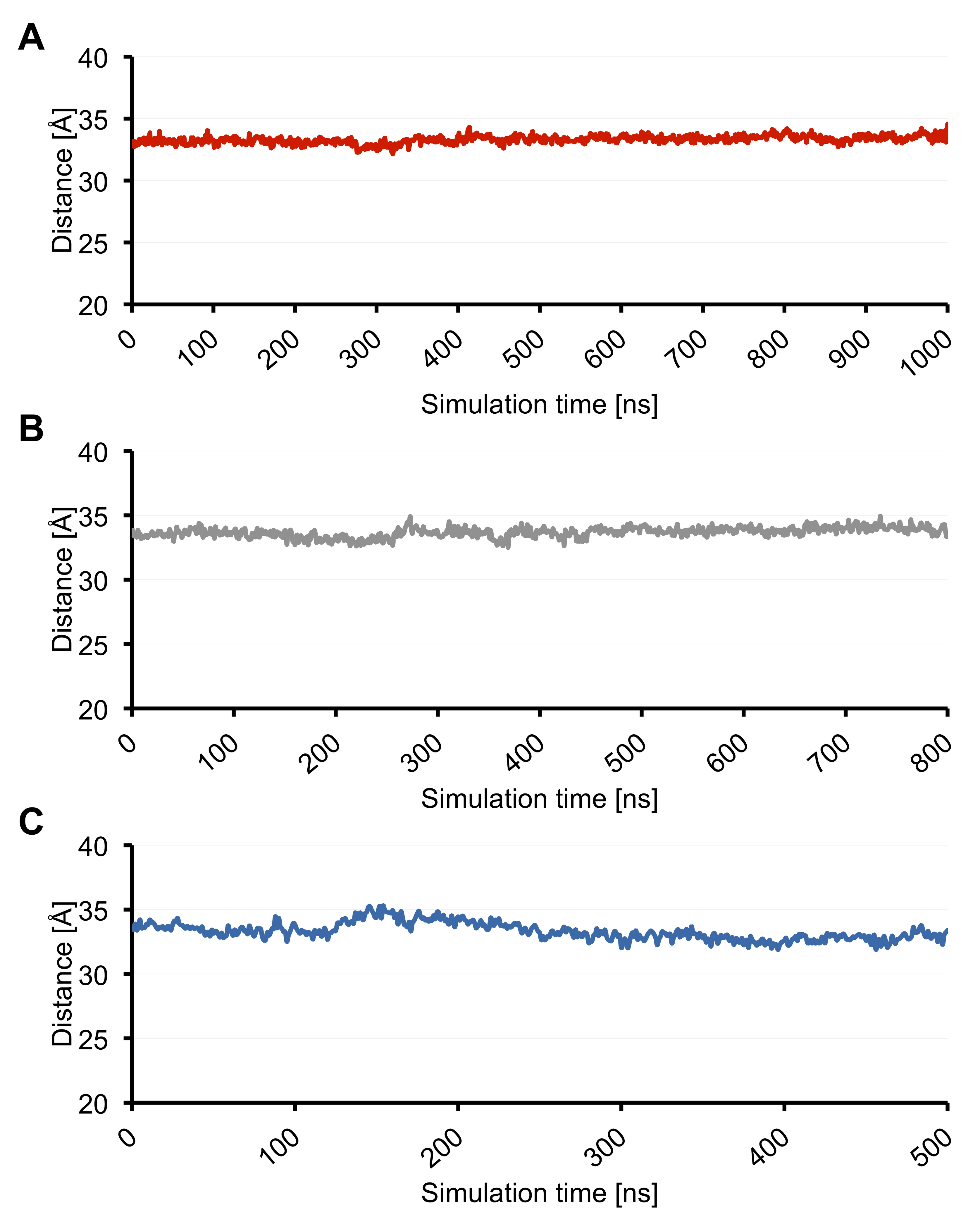

Supplement: Figure S4 — Distances between receptors and the C-termini of the G proteins. Distances between the centers of mass of D2R and the C-terminus of Gαi for the dopamine- (A), the aripiprazole- (B) and the FAUC350-complex (C) are shown in red, grey and blue, respectively. (TIFF) [file pone.0100069.s004.tiff]

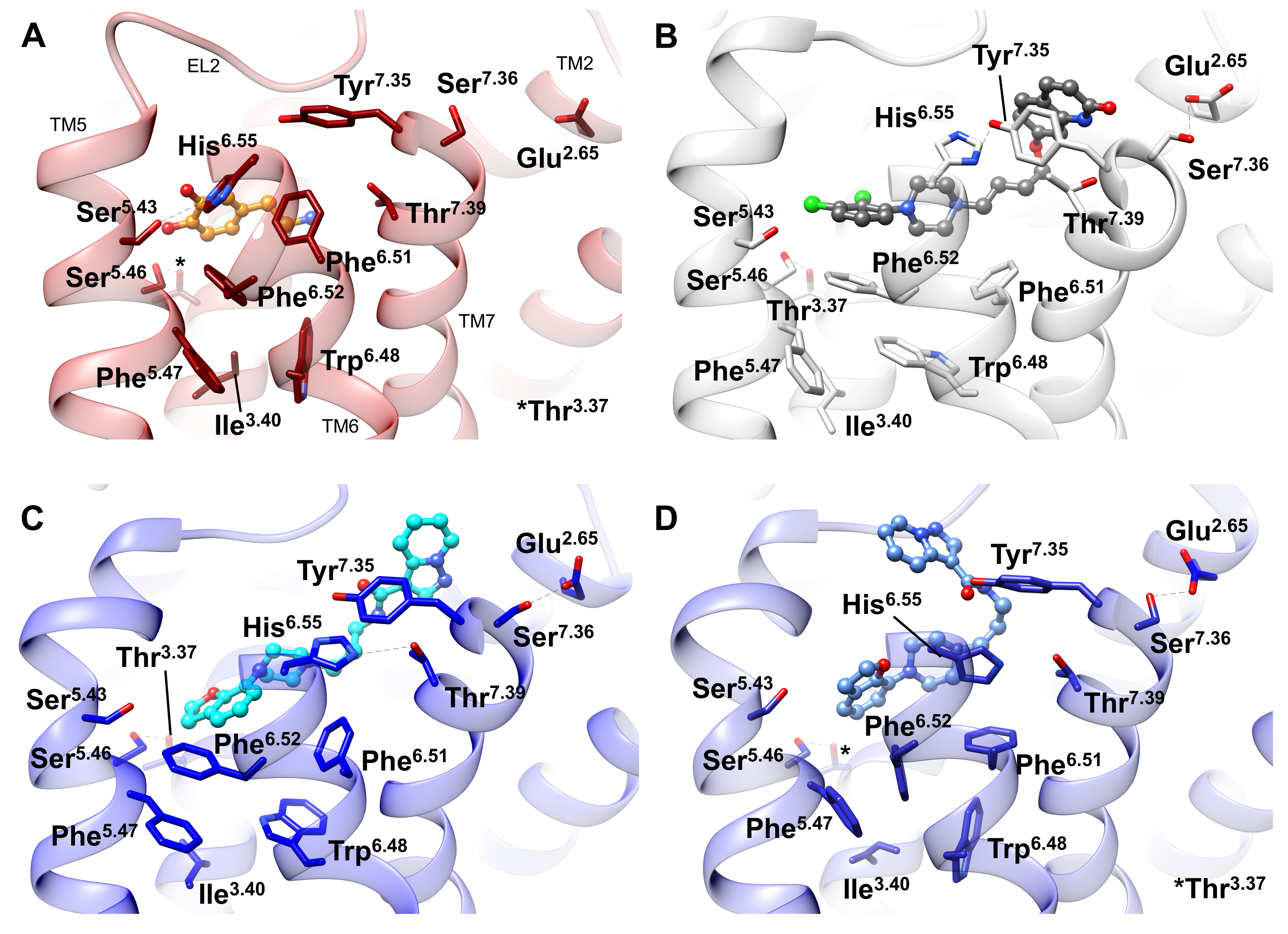

Supplement: Figure S5 — The binding pocket of the simulation systems. Side view into the binding pocket of the simulation systems. The backbone of D2R is shown as ribbons, important amino-acids stabilizing the conformation of the ligands are represented as sticks. (A) A representative snapshot of the conformation of dopamine (orange balls and sticks) within the binding pocket is shown. (B) A representative snapshot of the conformation of aripiprazole (dark-grey balls and sticks) within the binding pocket is visualized. (C, D) Representative snapshots of the conformation of FAUC350 within the binding pocket are highlighted, taken from within the last 20 ns of the simulation time (light-blue balls and sticks, C) and at 400 ns (blue balls and sticks, D). (TIFF) [file pone.0100069.s005.tiff]

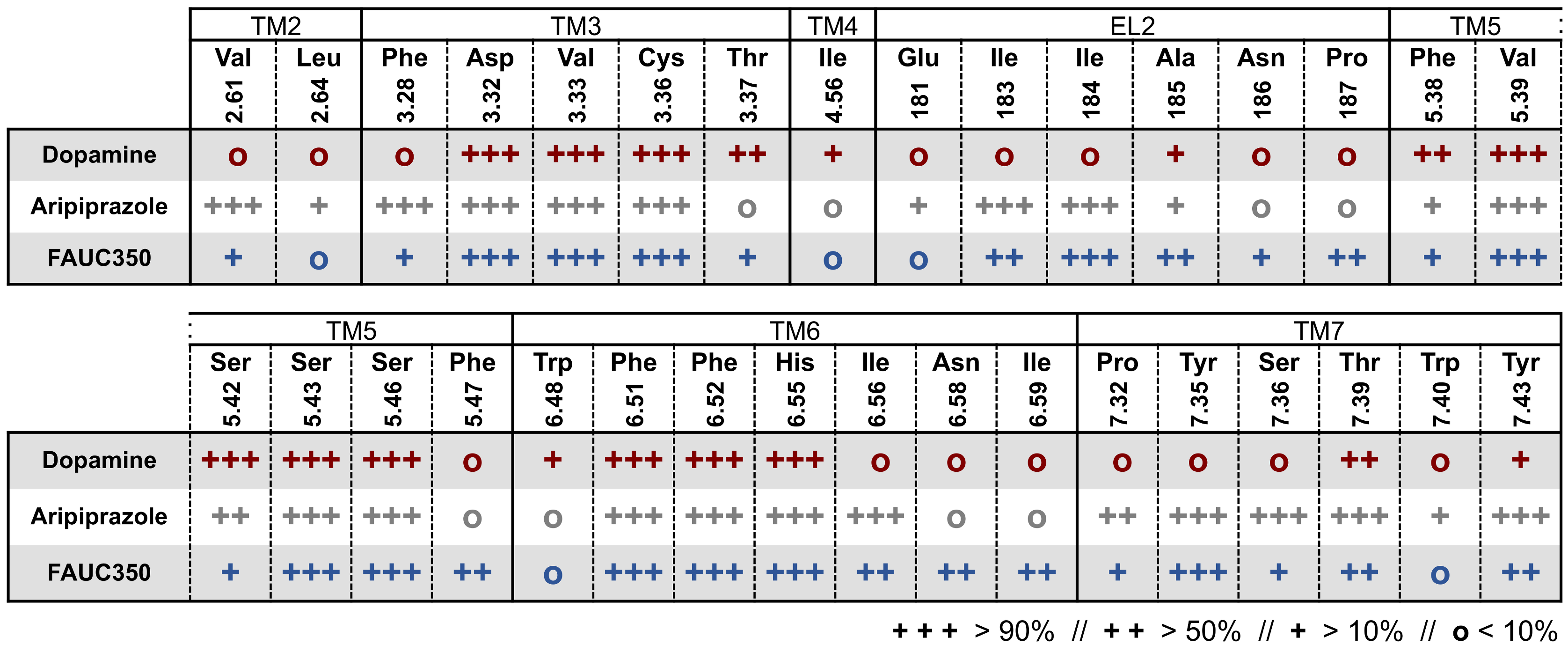

Supplement: Figure S6 — Residues within the binding pocket of D2R interacting with the ligands. A detailed contact analysis of residues within the binding pocket of the simulation systems interacting with the ligands is provided. An amino acid is considered as forming a contact to a ligand when at least one atom of the amino acid approaches at least one atom of the ligand closer than 3.5 Å. The contacts are investigated throughout the simulated time scales. (TIFF) [file pone.0100069.s006.tiff]

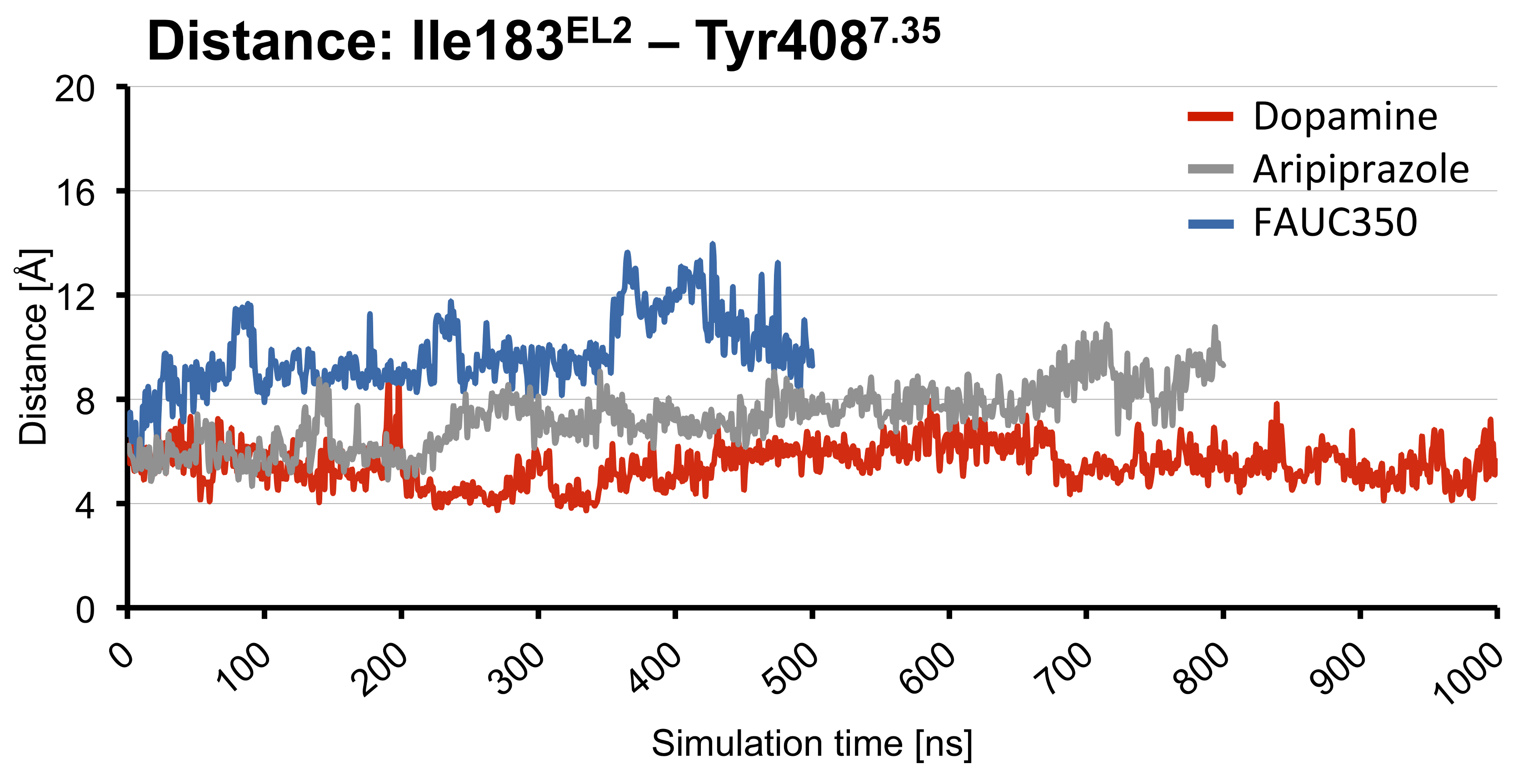

Supplement: Figure S7 — Distance between EL2 and the extracellular end of TM7. The distances between the side chains of Ile183 of EL2 and Tyr4087.35 of TM7 for the dopamine-, the aripiprazole- and the FAUC350-complex are shown in red, grey and blue, respectively. (TIFF) [file pone.0100069.s007.tiff]

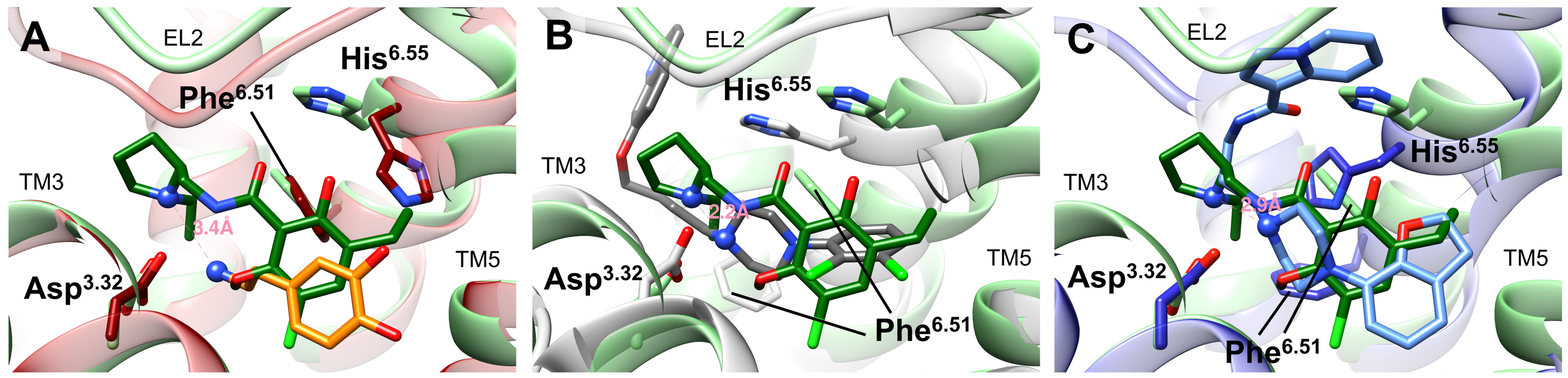

Supplement: Figure S8 — Comparison of the predicted binding modes of our agonists at D2R with the conformation of the antagonist eticlopride at D3R. Side view into representative snapshots of the binding pockets of D2R and the crystal structure of D3R. The snapshots represent average structures taken from 950–975 ns (dopamine), 750–775 ns (aripiprazole) and 350–375 ns (FAUC350). The backbone of the receptors is shown as ribbons, the ligands and important amino acids (Asp3.32, Phe6.51 and His6.55) stabilizing their conformation are represented as sticks. The positively charged nitrogen atoms of the ligands are highlighted as blue balls, whereas the distances between these nitrogen atoms of the ligands are given in light pink. The figure shows an overlay of eticlopride (green) at D3R with dopamine (orange/red, A), aripiprazole (grey sticks, B) and FAUC350 (blue, C) at D2R. (TIFF) [file pone.0100069.s008.tiff]

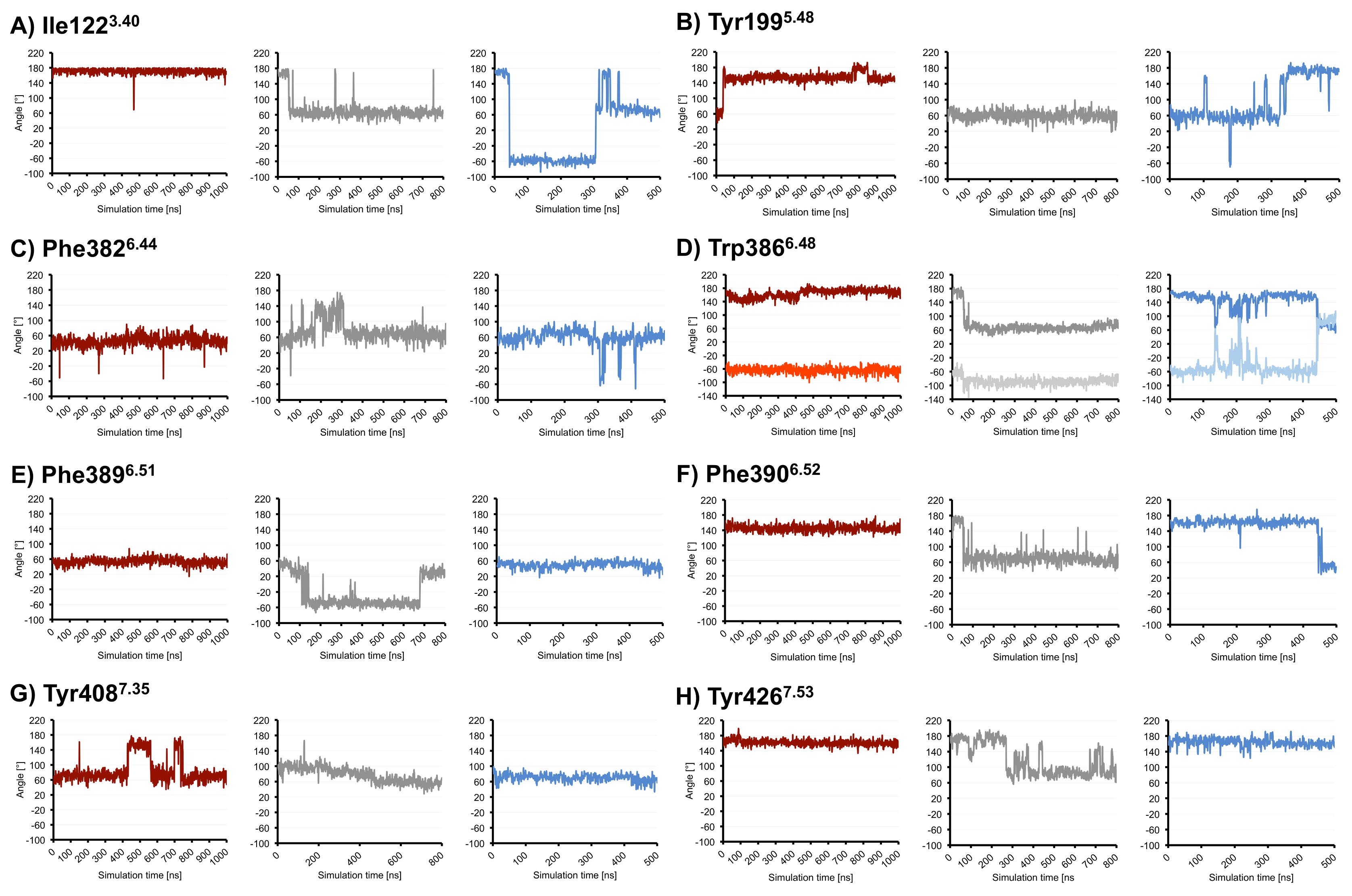

Supplement: Figure S9 — Ligand-specific dihedral angles of representative residues comprising the hydrophobic network between the ligand and the G protein binding pockets. (A–H) Dihedral angles (atoms: C-Cα-Cβ-Cγ) of important residues from the core of the hydrophobic network (Ile3.40, Tyr5.48, Phe6.44, Trp6.48, Phe6.51, Phe6.52, Tyr7.35, Tyr7.53), which connect the ligand and the G protein binding pockets of D2R, are shown as dark-red, dark-grey and dark-blue lines representing the dopamine-, the aripiprazole- and the FAUC350-complexes, respectively. In addition, the dihedral angle of residue Trp6.48 between atoms Cα-Cβ-Cγ-Cδ2 (D) is provided as light-red, light-grey and light-blue lines for the dopamine-, the aripiprazole- and the FAUC350-complexes, respectively. (TIFF) [file pone.0100069.s009.tiff]

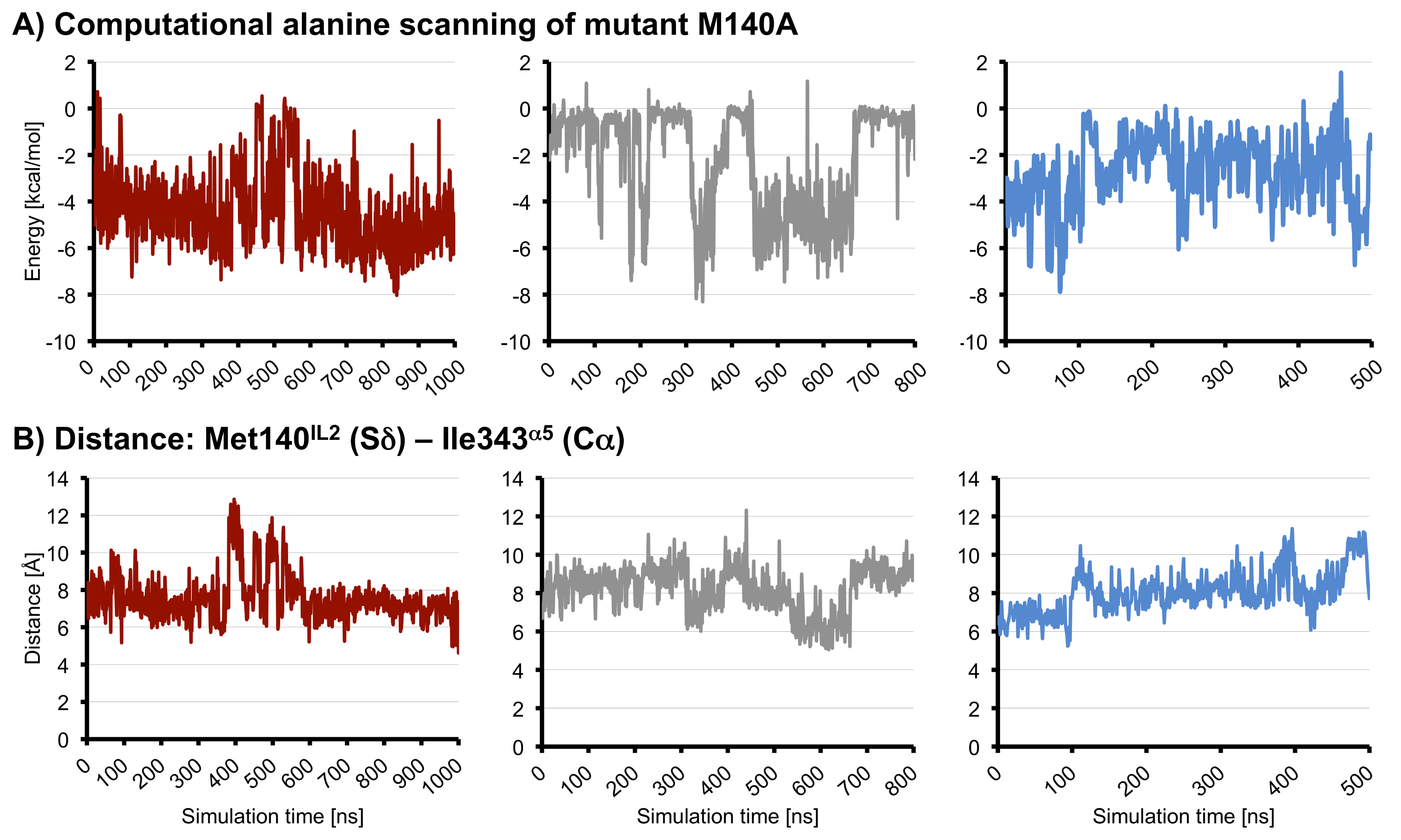

Supplement: Figure S10 — Investigations on residue Met140 of IL2. (A) A computational alanine scanning analysis for residue Met140 of IL2 is provided for the dopamine- (left), the aripiprazole- (middle) and the FAUC350-complexes (right). Whereas the M140A mutation within the dopamine-complex was connected to an impaired stabilization of the receptor-G protein interface, we observed weaker effects of this mutation within the aripiprazole- and the FAUC350-complexes indicating a less important role of Met140IL2 within the latter two simulation systems. (B) The distance between the side chain of Met140 of IL2 and the Cα-atom of Ile343 of α5 for the dopamine- (left), the aripiprazole- (middle) and the FAUC350-complexes (right) is shown. An increasing distance between these residues indicates a conformation of Met140 exhibiting reduced contacts towards the G protein. (TIFF) [file pone.0100069.s010.tiff]

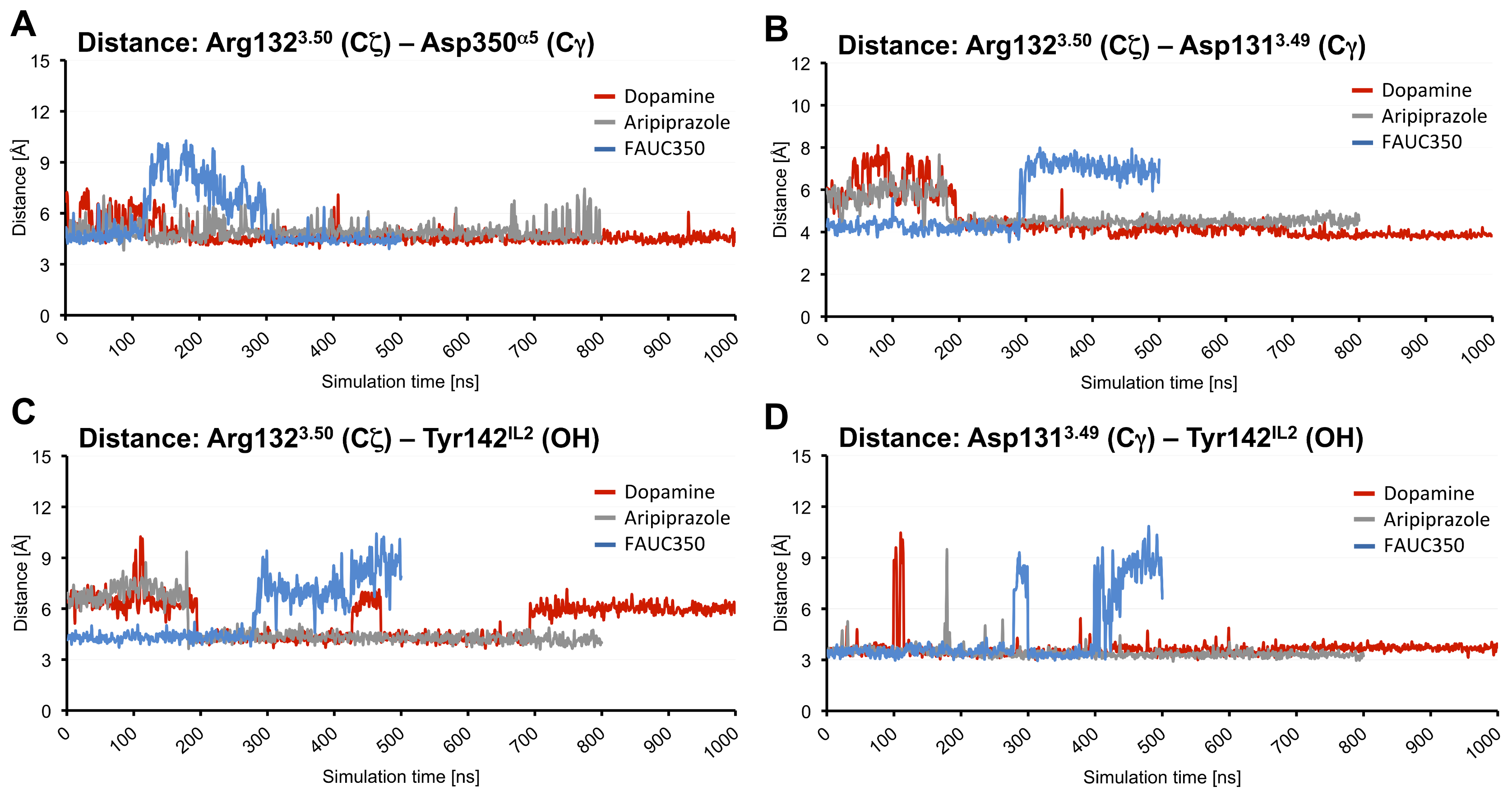

Supplement: Figure S11 — Distances between individual residues of TM3, IL2 and the α5 helix of the G protein. The distances between individual residues of TM3 (Asp3.49, Arg3.50), IL2 (Tyr142) and the α5 helix of the G protein (Asp350) are shown as red, grey and blue lines for the dopamine-, the aripiprazole- and the FAUC350-complexes, respectively. The distance between residues Arg3.50 and Asp350 of the G protein comprising a salt bridge (A), between residues Arg3.50 and Asp3.49 of TM3 (B), between residues Arg3.50 of TM3 and Tyr142 of IL2 (C) and between residues Asp3.49 of TM3 and Tyr142 of IL2 (D) is provided. (TIFF) [file pone.0100069.s011.tiff]

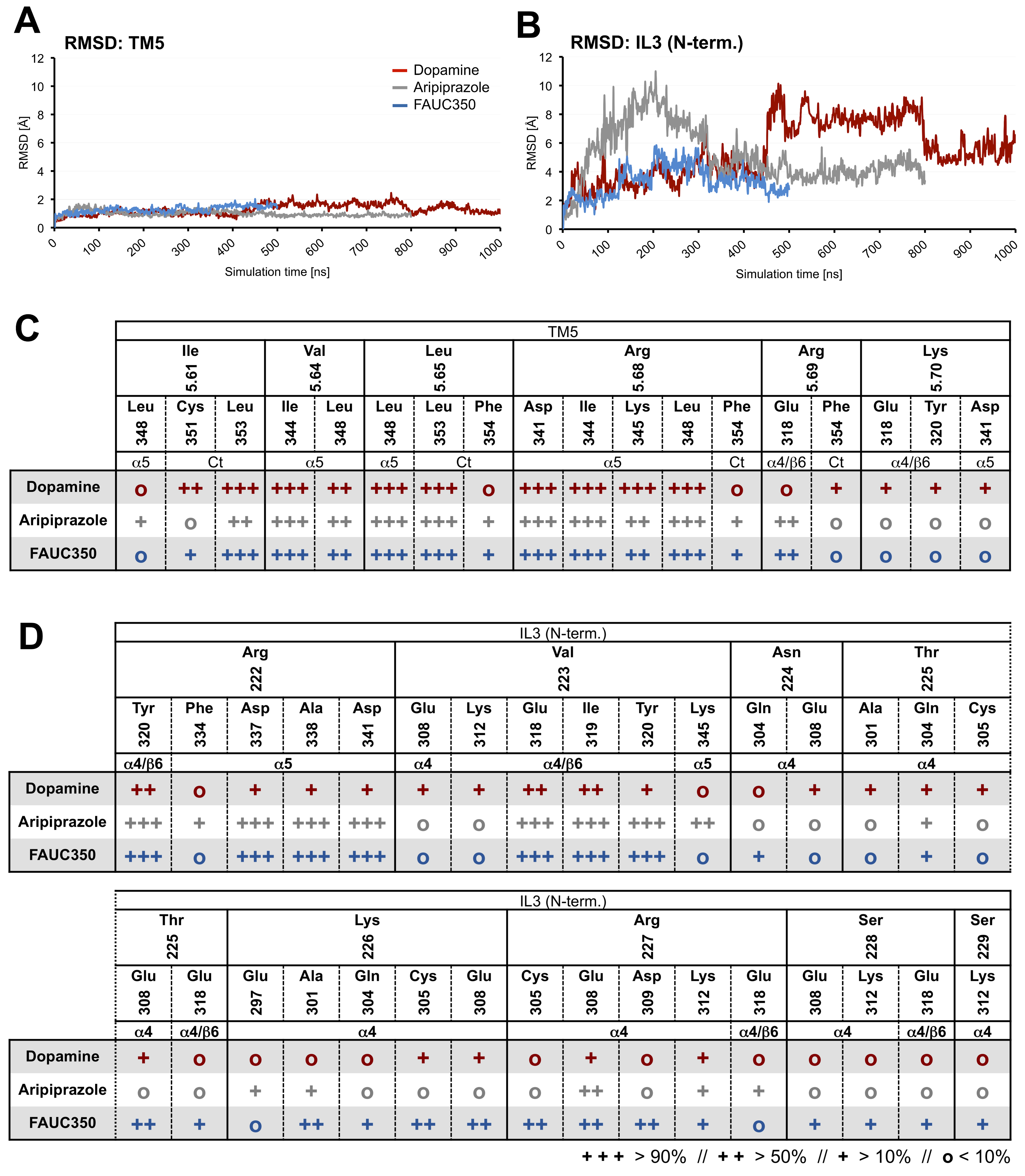

Supplement: Figure S12 — RMS-deviations and contact analysis of the TM5-IL3 region. (A) RMS-deviations for TM5 of D2R within the simulation systems are shown as red, grey and blue lines for the dopamine-, the aripiprazole- and the FAUC350-complexes, respectively. The values attribute a low conformational flexibility to TM5. (B) RMS-deviations for the proximal part of IL3 of D2R within the simulation systems are shown as red, grey and blue lines for the dopamine-, the aripiprazole- and the FAUC350-complexes, respectively. The values indicate a high conformational flexibility for IL3. (C, D) A detailed contact analysis between residues of TM5 and IL3 of D2R interacting with residues of the G protein is provided. An amino acid is considered as forming a contact when at least one atom of one amino acid approaches at least one atom of a second amino acid closer than 3.5 Å. The contacts are investigated throughout the simulated time scales. (TIFF) [file pone.0100069.s012.tiff]

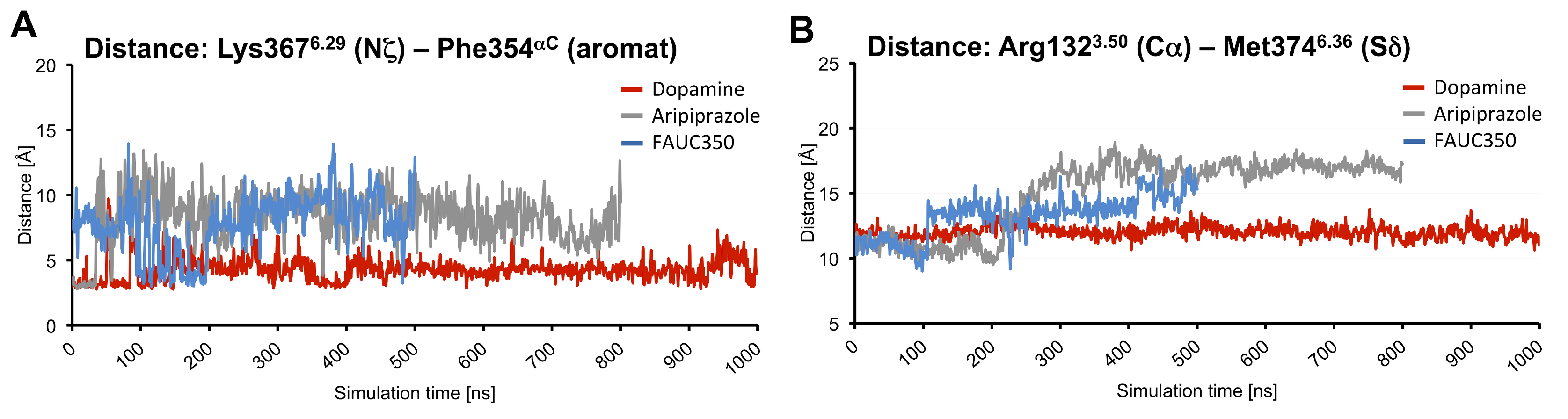

Supplement: Figure S13 — Investigation of TM6 residues Lys3676.29 and Met3746.36. (A) The distances between TM6 residue Lys3676.29 of D2R and the C-terminal residue Phe354 of Gα are shown. (B) The distances between D2R residues Arg1323.50 and Met3746.36 are shown. Values are represented in red, grey and blue for the dopamine-, the aripiprazole- and the FAUC350-complex, respectively. (TIFF) [file pone.0100069.s013.tiff]
