# Supplementary material for: Active-State Model of a Dopamine D2 Receptor - Gαi Complex Stabilized by Aripiprazole-Type Partial Agonists
Source: PLoS One. 2014 Jun 16;9(6):e100069. doi: 10.1371/journal.pone.0100069 (PMC4059746; doi:10.1371/journal.pone.0100069)
Supplement: Table S1 — Overview of the simulation systems and their simulated time scales. (DOCX) [file pone.0100069.s014.docx]

**Table S1.** Overview of the simulation systems and their simulated time scales.

|  | Ligand | Initial His393^6.55^ conformation | Total number  of atoms | Simulation time |
| --- | --- | --- | --- | --- |
| A | Dopamine | D2^Down^R | 224,760 | 1000 ns^a^  + 500 ns |
| B | Dopamine | D2^Up^R | 227,641 | 1000 ns^a^ |
| C | Aripiprazole | D2^Up^R | 227,577 | 800 ns |
| D | FAUC350 | D2^Up^R | 227,571 | 500 ns |

^a^previously published simulation: Kling RC, et al. (2013), PLoS One 8: e67244.
