# Supplementary material for: Active-State Model of a Dopamine D2 Receptor - Gαi Complex Stabilized by Aripiprazole-Type Partial Agonists
Source: PLoS One. 2014 Jun 16;9(6):e100069. doi: 10.1371/journal.pone.0100069 (PMC4059746; doi:10.1371/journal.pone.0100069)
Supplement: Table S2 — Chemical structures of the ligands investigated. (DOCX) [file pone.0100069.s015.docx]

**Table S2.** Chemical structures of the ligands investigated.

|  |  |  |
| --- | --- | --- |
| Dopamine | Aripiprazole | FAUC350 |
